# Recursive partitioning analysis of prognostic factors in WHO grade III glioma patients treated with radiotherapy or radiotherapy plus chemotherapy

**DOI:** 10.1186/1471-2407-9-450

**Published:** 2009-12-18

**Authors:** Chul-Kee Park, Se-Hoon Lee, Jung Ho Han, Chae-Yong Kim, Dong-Wan Kim, Sun Ha Paek, Dong Gyu Kim, Dae Seog Heo, Il Han Kim, Hee-Won Jung

**Affiliations:** 1 Department of Neurosurgery, Seoul National University College of Medicine, Seoul National University, Seoul 110-744, Korea; 2 Department of Internal Medicine, Seoul National University College of Medicine, Seoul National University, Seoul 110-744, Korea; 3 Department of Radiation Oncology, Seoul National University College of Medicine, Seoul National University, Seoul 110-744, Korea; 4 Seoul National University Hospital Cancer Research Institute, Seoul 110-744, Korea

## Abstract

**Background:**

We evaluated the hierarchical risk groups for the estimated survival of WHO grade III glioma patients using recursive partitioning analysis (RPA). To our knowledge, this is the first study to address the results of RPA specifically for WHO grade III gliomas.

**Methods:**

A total of 133 patients with anaplastic astrocytoma (AA, n = 56), anaplastic oligodendroglioma (AO, n = 67), or anaplastic oligoastrocytoma (AOA, n = 10) were included in the study. These patients were treated with either radiotherapy alone or radiotherapy followed by PCV chemotherapy after surgery. Five prognostic factors, including histological subsets, age, performance status, extent of resection, and treatment modality were incorporated into the RPA. The final nodes of RPA were grouped according to their survival times, and the Kaplan-Meier graphs are presented as the final set of prognostic groups.

**Results:**

Four risk groups were defined based on the clinical prognostic factors excluding age, and split variables were all incorporated into the RPA. Survival analysis showed significant differences in mean survival between the different groups: 163.4 months (95% CI: 144.9-182.0), 109.5 months (86.7-132.4), 66.6 months (50.8-82.4), and 27.7 months (16.3-39.0), respectively, from the lowest to the highest risk group (p = 0.00).

**Conclusion:**

The present study shows that RPA grouping with clinical prognostic factors can successfully predict the survival of patients with WHO grade III glioma.

## Background

Anaplastic astrocytoma (AA), anaplastic oligodendroglioma (AO), and anaplastic oligoastrocytoma (AOA) are defined as the major histological categories of WHO grade III gliomas, even though their classification based on the known molecular biology information remains controversial [1-4]. The relative survival rates at 5 years for AA and AO are 29.4% and 45.2%, respectively[5]. Although the increase is modest, standardized radiotherapy or chemotherapy has extended the survival period for patients with high-grade gliomas, suggesting the possible influence of prognostic factors such as age, performance status, symptom duration, tumor resection, histological type, and 1p/19q co-deletion [6-8]. However, most randomized trials pooled both grades III or IV astrocytic tumors and grade III oligodendroglial tumors as malignant glioma[7,8]. The only exceptions are the studies on PCV chemotherapy for AO[9,10]. A separate study of WHO grade III glioma is needed because of the observed difference in the outcome for grade IV glioblastoma using the same treatment protocols[11,12]. Even in those well-designed studies employing consistent treatment protocols, diverse biological behaviors and clinical outcomes clearly exist for patients with AA, AO, and AOA. Therefore, the prediction of survival outcomes for WHO grade III glioma patients based on known clinical prognostic factors attains importance with regard to the treatment plan.

Recursive partitioning analysis (RPA) classification, which was initially described by the Radiation Therapy Oncology Group (RTOG), is a useful tool that can divide patients into homogenous groups based on the length of survival[13]. RPA has an advantage over the proportional hazards model in identifying prognostic factors because it makes fewer modeling assumptions and has an established procedure that adapts to missing data through the use of surrogate measures[14]. Moreover, development of high-speed computer systems has made this tedious work easy for researchers[14]. The RTOG presented results from an RPA for all patients with malignant glioma (both WHO grade III and IV) throughout the duration of their clinical trials[13]. Results obtained for re-analysis of clinical trials using RPA, which focused only on glioblastoma patients, were also reported[14].

We evaluated the prognostic factors in the distinct group of newly diagnosed WHO grade III patients who were treated with radiotherapy or radiotherapy plus chemotherapy (PCV regimen) after surgery, in an attempt to predict survival outcomes by RPA. To our knowledge, this is the first study to address the RPA results focused specifically on WHO grade III gliomas.

## Methods

A total of 133 newly diagnosed AA, AO, and AOA patients were included in this single-center retrospective study. These patients were treated at the Seoul National University Hospital between January 1990 and December 2004, according to the baseline protocol, either with radiotherapy alone or with radiotherapy plus PCV chemotherapy. Histological diagnosis was re-evaluated according to the WHO 2000 classification. Patient data were collected according to the guidance specifications approved by the institutional review board and included information contained within the hospital charts and radiological studies. Clinical data such as age, performance status, extent of resection, and primary treatment modality after surgery were collected. Data that were unavailable in the medical records due to follow-up loss were obtained via a telephone interview with the patient or, if the patient was deceased, with his or her relatives with their permission.

The survival time was measured from the date of surgery to the date of the patient's death. Patients who were alive were classified as censored observations at the time of the last follow-up. Variables selected for prognosis analysis were those that were determined as significant based on previous reports[7-9,11,15]. Age was classified as 50 and above or under 50. Performance status was scored according to the Eastern Cooperative Oncology Group (ECOG) scales[16]. The extent of resection was categorized as a complete resection or incomplete resection (including biopsy only) based on the immediate post-operative imaging findings. Residual enhancing lesion on T1-enhanced images or measurable high signal intensity lesion on T2 images without enhancement of magnetic resonance images were considered residual lesion. Histological diagnosis and base-line treatment protocols were also included in the analysis.

The Kaplan-Meyer method was used to estimate the overall survival distributions. The log-rank test (level of significance α = 0.05) was used to test the differences in the overall survival distributions with respect to prognostic variables. A Cox proportional hazards model (level of significance α = 0.05) was used to adjust for covariates. These analyses were performed using SPSS^® ^ver 12.0. During RPA, free software (R version 2.6.2; rpart package version 3.1.39-1, http://www.r-project.org/) was used for the recursive decision tree creation with the split criteria of p < 0.01 in the log-rank test. The final nodes were grouped according to their survival times, and the Kaplan-Meier graphs are presented as the final set of prognostic groups.

## Results

The baseline clinical data of the study population are summarized in Table 1. Among 133 patients, 56 patients had a histological diagnosis of AA, 67 had AO, and 10 had AOA. The mean follow-up period of whole population was 88.8 months. The age distributions at the time of diagnosis, performance status, and baseline treatment were comparable among the groups defined by the histological diagnosis. A predilection for incomplete resection of tumors was apparent in the AA group due to their diffuse infiltrating nature of growth. Radiotherapy alone was part of the baseline treatment protocol before the mid-1990s (n = 67), and 6 to 12 cycles of PCV (procarbazine, lomustine, and vincristine) chemotherapy were added after radiotherapy beginning in the late-1990s (n = 66). In all patients, radiotherapy was initiated within 6 weeks after the surgery with a total mean dose of 59.4 Gy, 1.8 Gy per fraction with five fractions per week. The target volume included the residual tumor volume or surgical cavity and surrounding edema with a margin of 3 cm. Seven patients could not complete the planned dose of radiotherapy due to intolerance. PCV chemotherapy started within 4 weeks after the end of RT. Each cycle consisted of lomustine 110 mg/m^2 ^orally on day 1, procarbazine 60 mg/m^2 ^orally on days 8 to 21, and vincristine 1.4 mg/m^2 ^intravenous on days 8 and 29. Cycles were to be repeated every 6 weeks. The mean number of cycles completed per patient was 5.8. Due to the intolerance, 42% of patients were treated with less than 6 cycles of PCV.

**Table 1:** Baseline characteristics of the study population (number of patients).

	AA	AO	AOA	Total
	(n = 56)	(n = 67)	(n = 10)	(n = 133)
Age at diagnosis				
≥50 years	16 (28.6%)	17 (25.4%)	3 (30.0%)	36 (27.1%)
< 50 years	40 (71.4%)	50 (74.6%)	7 (70.0%)	97 (72.9%)
Performance status				
ECOG grade 0	5 (8.9%)	10 (14.9%)	2 (20.0%)	17 (12.8%)
ECOG grade 1	38 (67.8%)	44 (65.7%)	5 (50.0%)	87 (65.4%)
ECOG grade 2	9 (16.1%)	7 (10.4%)	0	16 (12.0%)
ECOG grade 3	2 (3.6%)	5 (7.5%)	3 (30.0%)	10 (7.5%)
ECOG grade 4	2 (3.6%)	1 (1.5%)	0	3 (2.3%)
Extent of resection				
complete	6 (10.7%)	27 (40.3%)	3 (30.0%)	36 (27.1%)
incomplete	50 (89.3%)	40 (59.7%)	7 (70.0%)	97 (72.9%)
Baseline treatment				
radiotherapy	31 (55.3%)	31 (46.3%)	5 (50.0%)	67 (50.3%)
radiotherapy plus PCV**	25 (44.7%)	36 (53.7%)	5 (50.0%)	66 (49.7%)

ECOG: Eastern Cooperative Oncology Group

PCV: procarbazine, lomustine, and vincristine

The median overall survival of the entire population was 59.0 months (95% confidence interval = 38.0 through 80.0). Estimated survival rates at 1-, 2-, 5-, and 10-years were 84.9%, 72.8%, 49.1%, and 35.5%, respectively. Survival was significantly better in radiotherapy plus PCV group (mean 118.0 months; 95% confidence interval = 100.7 through 135.4, median not reached) than radiotherapy only group (mean 57.7 months; 95% confidence interval = 41.7 through 73.8, median 29.0 months; 95% confidence interval = 19.2 through 38.9) (p = 0.00).

All possible prognostic variables determined as significant affected the overall survival in the univariate analysis. The results of the univariate analysis on median survival are as follows; age (65 months in < 50 years vs 19 months in ≥ 50 years), performance status (53 months in ECOG grade 1 vs 13 months in ECOG grade 2 (survivals showed stratification with ECOG grade and showed significant split between grade 1 and 2)), histology (29 months in AA vs 37 months in AOA vs 79 months in AO), and extent of resection (median survival not reached in complete resection vs 19 months in incomplete resection). The results of the Cox proportional-hazard analysis using these prognostic variables showed that the prolonged overall survival was independently affected by young age, good performance status, histological diagnosis of AO, complete resection, or addition of adjuvant PCV chemotherapy after radiotherapy (Table 2).

**Table 2:** Multivariate Cox proportional-hazards results for the prognostic value of variables related to the survival of WHO grade III glioma patients (n = 131).

	Hazard ratio	*p *value	95% confidence interval
Age ≥ 50 years	2.212	0.002	1.341-3.648
ECOG grade ≥ 2	2.179	0.003	1.305-3.638
AO	0.406	0.000	0.250-0.660
Complete resection	0.433	0.014	0.222-0.845
Radiotherapy plus PCV^†^	0.335	0.000	0.201-0.558

ECOG: Eastern Cooperative Oncology Group

AO: anaplastic oligodendroglioma

PCV: procarbazine, lomustine, and vincristine

**Figure 1:**
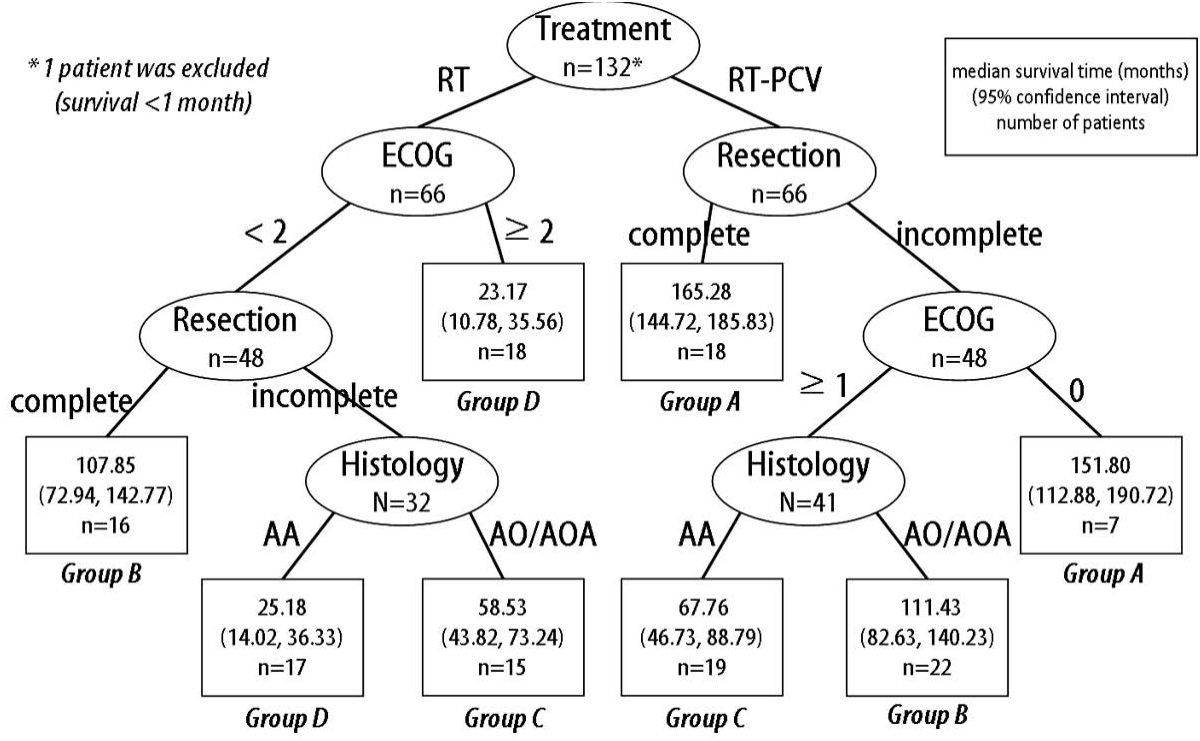
**Decision tree constructed by recursive partitioning analysis**. Terminal nodes (□) are categorized into 4 groups based on their median survival times.

**Figure 2:**
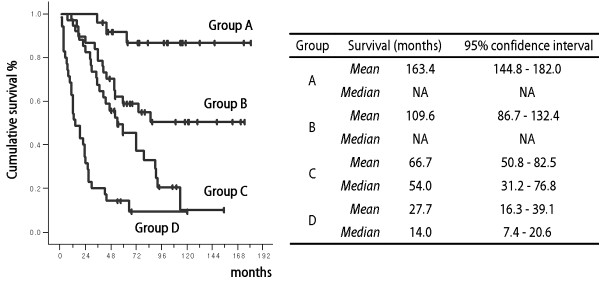
**Survival plot of the risk groups defined in Table 3**. Kaplan-Meyer analysis and the log-rank test revealed significant differences among the groups (p = 0.00).

Using the above mentioned significant prognostic variables, a recursive decision tree comprising 132 patients was created after exclusion of one patient during the analysis due to short survival time (less than 1 month). A total of 8 terminal nodes were produced in 6 splits (Figure 1). Among the variables included in the RPA, age was omitted from the split criteria according to the order of priority. Based on the median survival time of the terminal nodes, we were able to categorize them into four groups (Table 3). Survival analysis using the Kaplan-Meyer and log-rank test confirmed the significant differences among groups (p = 0.00, Figure 2). Independent of the histological diagnosis, patients treated with radiotherapy plus PCV chemotherapy after complete resection, or after incomplete resection but with the best performance status (ECOG grade 0), can expect the longest survival (Group A). Using the present results, survival probabilities can be estimated based on post-surgical clinical settings.

**Table 3:** Risk group splits according to the results of recursive partitioning analysis.

	Risk group	Number of patients	Number of events
Group A	RT-PCV, CR	25	3
	RT-PCV, ECOG 0, ICR		
Group B	RT-PCV, ECOG>0, ICR, AO/AOA	38	17
	RT, ECOG<2, CR		
Group C	RT-PCV, ECOG>0, ICR, AA	34	25
	RT, ECOG<2, ICR, AO/AOA		
Group D	RT, ECOG<2, ICR, AA	35	31
	RT, ECOG≥ 2		

RT: radiotherapy

PCV: procarbazine, lomustine, and vincristine

CR: complete resection

ICR: incomplete resection

ECOG: Eastern Cooperative Oncology Group

AO: anaplastic oligodendroglioma

AOA: anaplastic oligoastrocytoma

AA: anaplastic astrocytoma

## Discussion

Although AA, AO, and AOA are grouped as the same histological grade by the WHO classification system, among them exist diverse biological behaviors linked to clinical outcome, even within the same histological diagnosis. However, straightforward comparative analyses of the prognostic factors among the WHO grade III gliomas have been reported infrequently. In the present study, hierarchical stratification of prognostic variables, such as performance status, histological diagnosis, extent of resection, and adjuvant chemotherapy after radiotherapy among the WHO grade III gliomas could be deduced successfully using the RPA method. Based on the results of the present study, several conclusions could be drawn: (1) good complete resection is the most important prognostic factor for performance status, (2) the oligodendroglial component of the tumor favors better prognosis, and (3) PCV chemotherapy may be beneficial for certain groups of patients. These findings are not novel; however, using the risk group splits according to the given condition of the patients, we can estimate survival based on the chosen treatment modalities.

According to various studies, age is an invariably important prognostic factor of performance status for WHO grade III glioma[8,13,17,18]. We also observed significant prognostic values for performance status in the present analysis. However, it is important to note that age was excluded as a factor during the RPA, although it was a significant variable in the univariate analysis.

Analysis of the extent of resection for WHO grade III gliomas was based on the available data collected in an uncontrolled study setting. The present analysis has pitfalls, such as that superficial, small, well-demarcated tumors tend to undergo complete resection, whereas deep-seated, extensive, diffuse tumors are more likely to undergo biopsy only or incomplete resection[7,8]. Evidence for the prognostic impact of the extent of resection on AA remains sparse[19]; however, randomized studies investigating a combination of multiple treatment modalities support the beneficial impact of complete resection for AO and AOA[9,10].

Better prognosis of oligodendroglial tumors over astrocytic tumors is also supported by previous studies[20]. This evidence was further investigated, and the underlying genetic signature, such as the 1p/19q co-deletion in oligodendroglial tumors, was found to be responsible for the favorable prognosis[9]. Moreover, the diffuse nature of AA that precludes complete resection might have affected the outcome. It is a limitation of the present study that we could not include any molecular markers into RPA due to unavailability of appropriate tissue samples to carry on the analysis.

Radiotherapy and PCV chemotherapy, along with other alkylating agents such as nitrosoureas and temozolomide, are still considered to be the major options for the management of AA, AO, and AOA patients. Nonetheless, the role of radiotherapy in the treatment of AO/AOA and that of PCV chemotherapy for AA still requires further investigation [21-24]. Although the results of the present study show a beneficial effect of PCV chemotherapy on the outcome of the multivariate analysis, the possibility of a selection bias remains, since patients with poor performance status were excluded from further chemotherapy. Another noteworthy finding of the present study is the evidence for the diversity of the prognosis of AA, AO, and AOA patients, despite the best available treatment. The estimated survival ranged from good (group A) to bad (group D, such as glioblastoma) status. The results imply that AA, AO, and AOA patients with the best performance status, or with completely extirpated tumors, can be maintain prolonged survival without any recurrence with radiotherapy followed by PCV chemotherapy.

There are many clinical studies employing RPA to define risk groups[14,25-28]. RPA is a robust tool for the stratification of prognostic factors and for the identification of a homogenous group of patients for a given disease and treatment strategy. However, there are limitations to RPA application. For example, it is a post-hoc test, and no predictions can be made using the final splits, since prognostic factors are selected by chance[14]. If multiple variables that are highly correlated exist, the selection of factors may vary[14]. Despite these limitations, the RPA method allows for a clear distinction between the WHO grade III glioma patients risk groups. The advantage of this study is that only those patients with WHO grade III glioma who received the best available treatment were included in the analysis. It is more likely that the novel anticancer agent such as temozolomide will take over the mainstream of WHO grade III glioma management sooner or later. However, we believe that the systemized analysis for classic management has its own significance because it can be solid reference for the upcoming new therapeutic strategies.

## Conclusion

The present study shows that RPA grouping can successfully predict the survival of patients with WHO grade III glioma. Performance status, extent of resection, histological diagnosis, and treatment modality are the major determinants of patients' survival. These results may provide a tool for the collection of baseline data for further investigation of treatment modalities in the different risk groups of patients.

## Competing interests

The authors declare that they have no competing interests.

## Authors' contributions

CKP designed the study and wrote the draft and manuscript. SHL, JHH, and CYK were involved in the interpretation of the results. DWK, SHP, DGK, DSH, IHK, and HWJ managed the patients and reviewed the manuscript. All authors read and approved the final manuscript.

## Pre-publication history

The pre-publication history for this paper can be accessed here:

http://www.biomedcentral.com/1471-2407/9/450/prepub
